# Emergence of Functional Flexibility in Infant Vocalizations of the First 3 Months

**DOI:** 10.3389/fpsyg.2017.00300

**Published:** 2017-03-24

**Authors:** Yuna Jhang, D. Kimbrough Oller

**Affiliations:** ^1^School of Communication Sciences and Disorders, University of MemphisMemphis, TN, USA; ^2^Institute for Intelligent Systems, University of MemphisMemphis, TN, USA; ^3^Konrad Lorenz Institute for Evolution and Cognition ResearchKlosterneuburg, Austria

**Keywords:** infant vocalization, prelinguistic vocal development, language development, affect development, flexible communication

## Abstract

Functional flexibility, as manifest in the use of any word or sentence to express different affective valences on different occasions, is required in linguistic communication and can be said to be an infrastructural property of language. Early infant vocalizations (protophones), believed to be precursors to speech, occur in the first month and are functionally different from non-speech-like signals (e.g., cries and laughs). Oller et al. (2013) showed that infants by 3 months used three different protophone types with a full range of affect as manifest in facial expression, from positive to neutral to negative. These differences in affect were also shown to correspond to different illocutionary functions, unlike fixed signals, or vegetative sounds, which showed functional rigidity. The present study investigated whether infants show functional flexibility in protophones even earlier than the ages studied by Oller et al. (2013). Data were obtained from 6 infants across the first 3 months. Results showed that as early as the first month, infant protophones were already accompanied by variable facial affect valences and continued to be affectively flexible at the later ages. The present study thus documents the very early emergence of an infrastructural property of human communication.

## Introduction

### Overview

Before the emergence of speech, infants explore their vocal apparatus communicatively in prelinguistic vocalizations. When they produce sounds, they do not target consonants, vowels, words, or phrases. Instead they begin by producing more primitive sounds, protophones, precursors to speech. In using protophones infants build infrastructural capabilities that eventually lead to the emergence of speech. One of the critical infrastructural properties, called functional flexibility, differentiates protophones from cry, laugh, and vegetative sounds. The protophones include squeals, growls, and vowel-like sounds (hereafter “vocants”). The decoupling of sound and function/affect seen in protophones is analogous to (and forms a foundation for) the symbolic relations between words and meanings or between words and illocutionary forces. This decoupling contrasts sharply with the rigid association of cry with negativity and laugh with positivity. Previous research by Oller et al. (2013) illustrated that by 3 months (i.e., the fourth month) infants used at least three types of protophones to express a full range of affect (positive, neutral, and negative). The present work seeks to determine whether such flexible affect expression of protophones occurs earlier than 3 months.

### Functional flexibility

Any utterance in human language can be used to serve a variety of illocutionary functions such as acknowledgment, acceptance, joy, refusal, or seeking attention. For example, different illocutionary forces of an interjection, “oh” can be produced (a) in surprise when someone suddenly realizes something, (b) when someone hears something and expresses disappointment, or (c) when someone hears something wonderful. These three interjections might be accompanied by neutral, negative, and positive facial affect respectively. Functional flexibility can be measured as the degree to which a sound can be produced with differing communicative functions on different occasions of use. Cries, laughs, or hiccoughs cannot be said to be words in part because they do not have the degree of flexibility in serving illocutionary forces that words do: When someone hiccoughs during a meal, we know the hiccough is simply a bodily reaction to some digestive or respiratory condition—perhaps because the person ate too fast or drank too much or had some medical condition that caused involuntary contraction of the diaphragm. When an infant cries or laughs, we know the cry cannot be a positive signal and the laugh cannot be a negative one. The degree to which a cry or a laugh is associated with different communicative functions is very limited because in both cases the sound and the accompanying facial affect naturally constrain the perceivers' interpretation to either a positive state or negative state. In contrast, sounds like “oh” or any words in human languages have greater flexibility in their relations with communicative functions—any word can be produced in a positive, negative, or neutral state. Any language or precursor to language is required to have this property of functional flexibility, allowing speakers of language to be able to say any word with a variety of communicative functions, independent of circumstances.

The idea of communicative functions as we use it here comes from Austin's (1962) definitions of illocutionary and perlocutionary acts, and these have been adapted and extended by Oller and colleagues in studies of infant vocal development (Oller and Griebel, 2008; Oller et al., 2016) and cross-species comparisons (Griebel and Oller, 2008, 2014; Oller and Griebel, 2014; Griebel et al., 2016). The present study assumes this extended usage of Austin's terms. In this interpretation, an illocutionary force can sometimes consist of nothing more than the expression of an emotional state. In addition vocal exploration or vocal play can be portrayed as illocutionary forces. A second kind of function of communication pertains to the response of the receiver as a result of interpreting the sender's communication (including its illocutionary force, whether intentional or unintentional). The response of the receiver, also in Austin's terminology, is the “perlocutionary force” or “perlocutionary effect.”

In the present study we treat infant affect as the primary determiner of communicative function because in the neonatal period caregiver interpretations of affect play a major role in the perlocutionary effect of infant communications. Affect constrains the range of illocutionary forces that can be attributed to infant vocal communications to certain valence classes (positive, neutral, or negative) and similarly constrain the likely perlocutionary responses—positive affect may yield encouragement or praise, while negative affect may result in attempts to change the situation for the baby or may also result in scolding (Toda and Fogel, 1993; Oller et al., 2013). Positive affect in vocalization can be interpreted by caregiver/receivers as exultation, encouragement to continue interaction, and so on—positive illocutions. In contrast, negative affect can be interpreted by caregiver/receivers as rejection, complaint, or distress expression—negative illocutions. In keeping with the valence constraint, positive illocutions are constrained to remain within their valence class by their affect, and consequently, positive affect during vocalization cannot be interpreted as complaint, or another negative illocution. Thus, affect transmission (even if neutral) is a key to classifying the functions of a communicative act, especially in infants. For these reasons, we address early infant communicative functions by grouping them into valence categories (positive, neutral, negative) on the basis of affect. Note that our groupings and categorizations are always based on the perceptions of adult coders, and thus they represent interpretations by coders of infant affect or illocution, and there is no assumption in these judgments that infants have *intended* to communicate anything, nor that they have *intended* to associate any particular vocal type with any particular affect type.

Infants' ability to produce vocal types where there is no necessary coupling between form (e.g., vocal types) and function (i.e., illocutionary forces), as is manifested by spontaneous production of protophones with differing affect types, is foundational to the emergence and development of the speech capacity, and is required for word learning in later life. The association between a word, a phrase, or a sentence and its communicative function is always flexible, as exemplified earlier.

### The distinction between protophones and other vocalizations in infancy

Protophones, infant vocalizations that are neither vegetative, fixed signals, nor effortful grunts, are deemed precursors to speech for at least three reasons: (1) they can be produced spontaneously and endogenously (without any external stimuli), (2) they bear the property of functional flexibility, just as language does (for more information on protophones and their infrastructural properties, see Oller, 2000; Oller et al., 2016), and (3) they can be used flexibly in vocal interaction to respond to or initiate protoconversation (e.g., Stern, 1974; Trevarthen, 2001; Bigelow and Power, 2014). Each of the three infrastructural properties are foundational building blocks for language emergence (Oller et al., 2016).

Protophones occur very often in the first months. Nathani et al. (2006) found a strong tendency of very young infants to produce protophones. Even at 0–2 months, protophones substantially outnumbered cries. This predominance of protophones from the earliest months motivates interest in how these sounds help build foundations for speech. Further, the data showed a developmental increase in infants' production of speech-like utterances (protophones) in proportion to non-speech like utterances (i.e., cries, laughs, and vegetative sounds) across the first 20 months of life. The proportions of protophones increased from 65% of the sample of infant sounds for 0–2 month-olds to 95% for 16–20 month-olds. As yet unpublished results by Oller et al. (2014) suggest that even preterm infants still in the hospital at 32 and 36 weeks gestational age produced more protophones than cries.

A key question is why protophones occur so frequently from early in life, given that they seem so distant from speech. It has been speculated that these sounds provide a platform for development of speech and serve the immediate function of indicating state and well-being to parents (Locke, 2006). The human infant is altricial and thus needs long-term parental care and investment. Protophones appear to provide parents with useful clues about the likely survivability and reproductivity of the infant (Oller and Griebel, 2006).

If infants were very limited in their ability to produce protophones spontaneously and endogenously at birth, we presume they would also be unable to show functional flexibility in vocalization. Even newborns, within the first week of life, produce protophones spontaneously, according to longitudinal observations (Stark et al., 1975; Stark, 1981; Koopmans-van Beinum and van der Stelt, 1986; Oller, 2000). It appears that at the very beginning of life, infants do not produce these sounds with communicative intent, and there is usually no indication that the sounds are directed to anyone. During the weeks after birth, parents sometimes notice that their infants produce sounds as vocal play (that is, for no obvious reason other than the interest of the sensations experienced in vocalizing), and that such vocal play activity can occur when infants are all alone. Locke (2006) argued that spontaneous vocalization may be a signal of well-being for parents, and thus when it occurs in the course of development, it may elicit commitment to long term care. Spontaneous playful actions observed in animals have also been argued to function as signals of well-being and can serve as an especially useful index of physical and psychological well-being in young primates (Mason, 1965). Thus, functional flexibility of infant protophones does not necessarily imply that infants *intend* to produce protophones in association with particular states of affect.

By 3 months, the protophones differentiate into at least three types (Buder et al., 2008): (a) vocants generally produced in the mid-pitch range (approximately 250–400 Hz) of each infant using a pattern of vocal fold vibration called normal phonation; this is the phonatory type that occurs overwhelmingly in syllables of speech, (b) squeals with high pitch (typically at least twice as high as the infant's mid pitch), often produced in loft or falsetto phonation, and (c) growls, which have either low pitch (typically half or less than the mid-pitch value), often with fry or “pulse” phonation, or noisy dysphonation (Stark et al., 1975; Oller, 1980; Holmgren et al., 1986). Audio-video examples of all three phonatory protophone types can be found at babyvoc.org, IVICT, and in Oller et al. (2013, Supporting Information Appendix), and in the Supplementary Material to the present paper. Additionally, there are less frequently occurring protophones, which will be termed “other protophones” in the present paper and which will be described below^1^.

It is important to emphasize that while acoustic differentiations of some sort must underlie the auditory identification of protophones and other infant sounds, our analyses of functional flexibility rely on auditory judgments of vocal type rather than acoustic ones. The reason is fundamental: The development of the human infant's vocal communication must be guided by caregiver responses and elicitations. These parental actions are dependent upon auditory judgments of protophone types (which parents often imitate or elicit) as well as judgments of cry and laughter, and on visual judgments of infant facial affect. To the extent that parental reactions to these sounds and expressions of affect drive the development of the functionally flexible infant communication system, it is precisely the caregiver perceptual judgments (simulated by laboratory listeners) that are the natural target of our coding. Acoustic analysis thus plays no role in the categorization by the coders in the present study. The role of acoustic analysis is only in helping to elucidate possible bases for the intuitive judgments about infant vocal types made by human listeners. Buder et al. (2008) and subsequent work suggest that protophone judgments are complex, including at least pitch (f0), amplitude, and spectral factors. We are continuing to pursue acoustic analysis to elucidate the bases for identifications of infant vocalizations by human listeners.

### Empirical research on functional flexibility in early human communication

Seeking roots of language in prelinguistic communication, scientists have sought to identify affective or communicative content in the production of infant vocalizations (Oller, 1981; Stark and Bernstein, 1993; Papaeliou et al., 2002; Scheiner et al., 2002; Iyer and Ertmer, 2014). Very few have, however, considered the relation between prespeech sounds and speech in terms of infrastructural properties of human language, and how these properties of protophones lay foundations for the emergence of speech. Even fewer have used quantitative methods to explore these infrastructural properties developmentally.

Quantifying functional flexibility of infant protophones, Oller et al. (2013) demonstrated that protophones were very distinct from cries or laughs, which were consistently associated with either positive or negative affect. The protophones bore the property of functional flexibility, like words, or sentences in language. Cries and laughs, for example, were scarcely judged to be neutral in facial affect (mean = 4% of all cries and laughs were deemed neutral facially), whereas infant protophones (i.e., with flexible functions like those of language) showed predominant neutrality in facial affect (mean = 64%), allowing for flexible association with a variety of other communication functions that are consistent with neutral valence such as requesting, solitary vocal play, or designation.

Oller et al. (2013) further fleshed out the idea of functional flexibility by examining the following six patterns of how protophones produced by infants in the first year of life were significantly different from cries and laughs: (1) protophones were more often positive (in facial affect) than cries; (2) protophones were less often positive than laughs; (3) protophones were more neutral than either cries or (4) laughs; (5) protophones were less negative than cries, and (6) protophones were more negative than laughs. All six patterns were confirmed with highly significant odds ratios and showed large effect sizes.

### The purpose of the current study

The present study aims to extend Oller et al. (2013)'s prior effort with a focus on infants' first 3 months of life, an age range that was not addressed in the study. First, the present study sought to determine if functional flexibility can be identified by laboratory coders in infant vocalizations of the first 3 months. This question was asked in order to determine if functional flexibility emerges even earlier than reported in the prior paper. As in the prior paper, the coders were blind to the purpose of the study at the time of coding. We asked, “at what age can functional flexibility first be discerned?” Also as in the prior paper, coders judged affect and vocal type independently, in video-only for affect, and in audio-only for vocal type.

Second, we examined whether functional flexibility can be further demonstrated in other protophone types (i.e., raspberries, other consonants alone, and ingressive sounds) in addition to the three primary ones that are defined by phonatory properties (squeals, growls, and vocants). The prior paper did not include analysis of the “other protophones.” However, given the common occurrence of other protophone types in some infants, we considered it important to include them in this and future analyses.

Here are the present hypotheses:

- Functional flexibility will be discernible for protophones in the first 3 months: We predict significant odds ratios, showing protophones to be accompanied by less negativity than cry and greater neutrality than cry.
- Functional flexibility will be discernible in “other protophones,” just as in the phonatory protophone types (squeals, vocants, and growls): We predict functional flexibility in the other protophones will also be indicated by significant odds ratio differences.

## Methods

### Selection of participants

A written consent form and a simple questionnaire were completed by the infants' parents before any recordings for the longitudinal research project on infant vocal development from which the recordings for the present study were drawn. Inclusion criteria required subjects to have no language, hearing, or developmental disorders. All procedures were approved by The University of Memphis Institutional Review Board for the Protection of Human Subjects.

From this longitudinal research, we selected all six American mother-infant dyads who had at least 1 recording day (approximately 1 h of data) for 0 and 1 months and at least 2 recording days at 2 months as indicated in Table 1. In some cases these recordings were made in one continuous hour. But usually there were interruptions due to infant physical discomforts, resulting in persistent crying. In such cases the recordings were resumed on the same day, usually within an hour, after feeding, changing, or comforting the infant. These interruptions caused the recordings indicated in Table 2 to be broken up into “segments” often considerably shorter than 1 h, many of them about 20 min in duration.

**Table 1:** **Number of 1 h recordings available**.

**# of ~1 h recordings available**			
**Infant ID**	**0 month**	**1 month**	**2 month**
Infant1	2	1	2
Infant2	1	1	2
Infant3	1	1	2
Infant4	1	1	2
Infant5	1	1	2
Infant6	1	1	2
Total	7	6	12

**Table 2:** **Duration and number of utterances of recording segments for repeat-observation coding**.

**Infant**	**Age**	**Duration (in min)**	**# of utterances located**
Infant 1	0	21	96
	1	20	97
	2	20	108
Infant 2	0	16	143
	1	21	103
	2	26	128
Infant 3	0	20	117
	1	20	145
	2	20	138
Infant 4	0	20	157
	1	20	101
	2	20	136
Infant 5	0	20	148
	1	20	114
	2	20	156
Infant 6	0	21	111
	1	20	107
	2	26	163
Mean duration and # of utterances per recording segment		20.61	131.56
*SD*		2.23	38.72

The recordings had been made for the longitudinal study in a laboratory designed to resemble a child's playroom, with eight cameras positioned in the corners of the room, one high on the corner, and one low, in each case. From an adjacent control room an experimenter chose for recording two of the eight possible video channels and switched as needed to obtain a view of the infant's face along with another view of the interaction between the infant and the parent and/or experimenters throughout the recording. Both infants and parents wore wireless microphones with signals digitized at 44 kHz.

The infant, of course, was always present in the hour-long recordings, which at different points during the hour included parent-infant interaction, an interview of the parent with the experimenter, and periods of silence from the adults, allowing the infant to vocalize or bid for interaction in any other way. The segments selected for the present work were always from either the interaction or interview circumstances, during which infants were often very vocally active.

### The coding plan and software

The present research was intended to examine functional flexibility of infant vocalization in the first year. Consequently coding for the primary data collection was conducted in a way similar to that of Oller et al. (2013), with both vocal type and facial affect being coded on separate passes using repeat observation (repeat listening for vocal type and repeat viewing for facial affect). Repeat-observation coding is, however, very time intensive. Efficient allocation of coding staff time required focusing the repeat coding on one approximately 20-min segment for each infant and age (18 such segments).

To locate 20-min periods from the recording days during which there was considerable vocal activity to code, we began by having one group of coders work in real-time to locate periods of high volubility (number of infant vocalizations). All the recording material was thus coded in real-time by this first group of coders in order to enable the 20-min segments to be selected efficiently. After selection of the eighteen 20-min segments, a separate group of observers coded in repeat observation to provide the primary data for the study.

All the coding was conducted in the same software environment used in Oller et al. (2013) [Action Analysis Coding and Training software, AACT (Delgado et al., 2010)]. AACT coordinates frame-accurate video and audio presentation with real-time acoustic displays in TF32 (Milenkovic, 2001) and allows convenient location of utterances for coding with keystrokes or mouse control. Two channels of video can be presented simultaneously. For details on the software and other recording details, see Supporting Information Appendix to Oller et al. (2013).

### Real-time coding to locate periods of high vocal activity in the recordings

As training for real-time coding, four graduate students in Communication Sciences and Disorders at the University of Memphis were presented with a lecture by the second author on vocal type coding for the above listed categories. During the lecture we presented real examples of previously coded infant utterances, all of which either met a consensus standard for one of the vocal categories or illustrated ambiguities of possible coding judgments based on prior listening experience in the laboratory. The infant vocal types in question are graded rather than discrete, and consequently even though there are many utterances that pertain to a single category unambiguously, there are other utterances that are judged to possess features of more than one of the categories. The coders were trained to focus on the most perceptually salient features of the utterances and to base their judgments on those most salient characteristics. Examples of the three primary protophone types produced by a 0-month-old infant from the present study are provided along with acoustic displays in the Supplementary Material.

The coders also passed the training modules of our on-line infant vocalizations training system (IVICT, Infant Vocalization Interactive Coding Training, at babyvoc.org) that has been developed to facilitate both laboratory training and training of parents in categorizing infant vocalizations with a common terminology (cry, squeal, raspberry…). The training on infant vocal types can be fairly brief because the categories correspond to naturally recognizable types, commonly reported by parents of infants in the first year of life when presented with an open-ended question such as “what kinds of sounds does your infant produce?” Thus, the primary point of training is simply to ensure that all the coders use the same terms to refer to the categories and that they make their judgments intuitively.

In a final stage, coder agreement was assessed based on coding of recordings drawn from each of the infants included in the present study, with examples drawn from all the vocal types to be coded. Background on the coding scheme, along with extensive details regarding training requirements, acoustic characteristics of the primary protophone types, reliability of acoustic identification for those protophone types, the tendency of infants to produce the individual protophone types repetitively, and coder agreement on the protophone types are provided in the Supporting Information Appendix to Oller et al. (2013).

The real-time task was to code infant vocal types as either vocant, squeal, growl, other, cry, or laugh in real time for all the segments of the recordings of six infants at three ages (Table 1). Coders tapped their responses on the keyboard as they listened to infant recordings without any stopping. Whenever they heard an infant utterance, they needed to enter one and only one code for the utterance immediately upon hearing it.

The real-time coding was conducted independently by each of the first group of four coders, with both video and audio playing during every coding session. The first author collated the results and located the first 20-min segment at each age that met the requirement of at least 96 infant utterances according to the real-time coders. Table 2 lists the durations and numbers of utterances for the segments that were selected and then submitted to repeat-observation coding.

### Primary coding for the present study, in repeat-observation mode

Before repeat-observation coding could begin, the first author located infant utterances within each recording segment that had been selected based on volubility from real-time coding (Table 2) and placed boundary cursors for them in AACT. The boundaries were determined using a breath-group criterion (Lynch et al., 1995) for both protophones and cries/laughs so that the durations of protophones or cries/laughs were both based on the same principle—one utterance per expiration. Having bounded the utterances, the list of the utterances was available in AACT to coders so that they could click on each utterance location on the list, one by one, and the program would jump to each location so that the sound or facial affect could be coded immediately, skipping all intervening material. This is different from real-time coding where the observer experiences the entire context of each utterance.

A second group of four graduate students in Communication Sciences and Disorders at the University of Memphis received similar training as the four real-time coders for all the same vocal types and then were assigned as repeat-observation coders. Their task included vocal type coding, conducted with audio only (video was closed), and facial affect coding, conducted with video only (audio was muted). For facial affect, the observers were instructed to code each utterance (actually just the period of time during which the utterance occurred, since audio was muted) as positive (smiling), negative (frowning or grimacing), neutral (neither smiling nor frowning), or “can't see” in cases where the infant's face was not visible in either of the two camera views. Eight percent of the utterances were dropped from the final analysis due to a report of “can't see” by at least one coder.

Each one of the repeat-observation coders received a different counter-balanced order of all 18 sessions and coded them independently, once for facial affect (video only) and again separately for vocal type (in audio only). The primary author also coded in repeat observation, so there were 5 coders altogether, each coding 18 sessions twice (once for vocal type and once for facial affect), for a total of 180 coding sessions.

We used kappa statistics (Cohen, 1960; Landis and Koch, 1977) and Pearson correlations to evaluate coder agreement for vocal type and affect judgments on the 18 segments. The assessment of agreement provided both a methodological measure and a measure of the degree to which the “categories” of vocal type and facial affect are recognizable and distinguishable. It is important to remember that such categories are overlapping rather than discrete, representing continuous dimensions of acoustic and visual information that are used as information by caregivers about infant state and developmental level. Consequently kappa agreement values are always expected to be modest.

The mean kappa agreement between the coders and the first author was 0.49 for all the vocal types coded (i.e., moderate agreement for vocant, squeal, growl, “other protophones,” and cry). However, the primary focus of the functional flexibility study is the binary contrast of protophones vs. cry. The mean kappa agreement for protophones vs. cry was 0.68 (substantial agreement). Kappa agreement was 0.65 for facial affect (substantial agreement for positive, neutral, negative). Mean intercoder correlation assessed at the session level was 0.93 for vocal type coding (numbers of utterances judged to be protophones for all five coders) and 0.90 for facial affect (numbers of utterances judged to be neutral for all five coders) (*N* = 18 sessions).

In order to ensure unimpaired hearing and seeing of the stimuli during repeat-observation coding, the utterances were played in such a way that the boundaries were “stretched” to include the 50 ms before and the 50 ms after each utterance. This precaution eliminated rise-time anomalies for utterances and ensured that the visible periods would include all video frames pertaining to the utterances. In both facial affect and vocal type coding the observers were allowed three listening or viewing opportunities for each utterance.

The 20-min segments contained a mixture of two circumstances: parent-infant vocal interaction (mother talking with baby) and interview (mother talking with experimenter). Because functional flexibility of infant vocalization has been shown to occur in similar degrees for both these circumstances (see Oller et al., 2013, Supporting Information Appendix, Robustness of functional flexibility of protophones across contexts), we anticipated no important differences in our results across the circumstances.

### Data analysis

As in Oller et al. (2013), odds ratio analyses were conducted to assess predictions regarding positivity, negativity and neutrality of facial affect associated with each of the vocal types. Instead of using all 6 hypotheses of the prior paper, however, we used 3, eliminating those related to laughs, because at the early ages focused on in the present study, infants rarely produce laughs. Indeed, very few laughs were indicated as vocal types by the coders—only 8 laughs among the 2268 infant vocalizations in the sample.

## Results

The total number of utterances produced by the six infants in the 20-min segments was 772 for 0 months, 667 for 1 months, and 829 for 2 months (including both protophones and cries). The 0-months data composed around 34% of the dataset, the 1-months 29%, and the 2-months 37%. Individual infants contributed 13–18% the utterances to the final dataset (*N* = 2,268 utterances). In the Supplementary Material, we supply a Summary Table indicating the mean number of utterances across the 5 coders cross-classified by facial affect and vocal type across the three ages.

### Descriptive overview of the present study in comparison with results of Oller et al. (2013)

**Figure 1:**
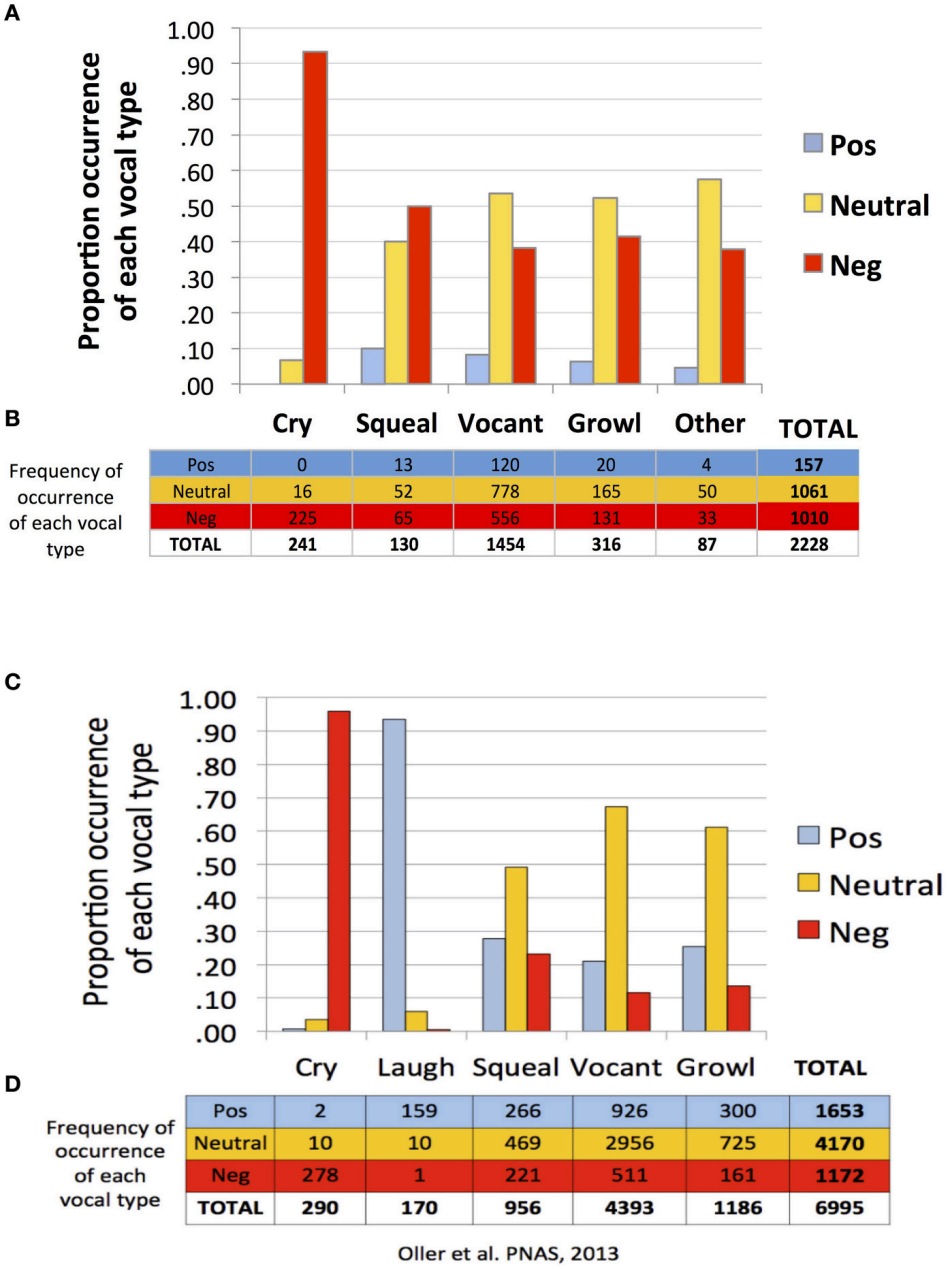
**Functional flexibility of protophones in the present study and in Oller et al. (2013)**. The data in **(A–D)** are based on a single coding of each utterance. The data from Oller et al. (2013), panels **(C,D)**, are based on a single consensus coding for each utterance. To maximize comparability, the present data as displayed **(A,B)** are from a single coder, the one who was most experienced among the five coders. Both data sets **(A–D)** show functional flexibility, but they differ in that positivity was less common and negativity more common in the present data. Regardless of which coder's data from the present study is used for comparison, the pattern of results is similar to that displayed in **(A,B)**.

Similar to Oller et al. (2013), findings of the present study suggest a strong tendency toward functional flexibility in the protophones but not in cry. Figure 1 shows that cries were overwhelmingly associated with negative facial affect, according to the coding of the recordings, in both the present study of infants 0–2 months of age (first panel, A and B) and in Oller et al. (2013) of infants 3–11 months (second panel, C and D). In both studies the protophones showed considerable proportions of utterances associated with non-negative (neutral or positive) facial affect. However, the protophones in the present study showed lower proportions of positivity and higher proportions of negativity of facial affect than in the prior study. Only about 7% of the utterances in the present study were deemed facially positive, in contrast with 24% in the prior one. The low proportion of positivity here is clearly associated with the fact that very young infants smile and laugh very little, with laughter generally not appearing with consistency until 3 or 4 months (Sroufe and Wunsch, 1972).

Neutral affect during vocalization may be thought of as an indicator of voluntary control, and indeed a great deal of mature speech is produced with neutral facial affect. But again the 0–2 month olds of the present study showed less neutrality of protophones than the older infants of Oller et al. (2013). Among the protophones, squeals showed least neutrality (40%) and most negativity (50%) and positivity (10%). In addition, vocants occurred most frequently among all the vocal types (number of vocants = 1,454, more than 65% of the utterances) both in this dataset and that of Oller et al. (2013) (number of vocants = 4,393, more than 63% of the data).

### Statistical results for the hypotheses

We examined the first hypothesis by considering the three predictions of Oller et al. (2013) regarding functional flexibility of cry and protophones using odds ratios. The analyses assessed three predictions for each vocal type—vocant, squeal, and growl. These three protophones were expected (as in the prior study) to show the following three patterns: (1) greater neutrality of facial affect than cries, (2) less negativity of facial affect than cries, and (3) greater positivity of facial affect than cries. These three predictions were assessed at all three ages for each of the three protophone types, so that there were 27 individual predictions to assess statistically under hypothesis one (Table 3). An additional 9 predictions are reported for “other protophones” in the table, predictions that will be considered below under hypothesis two, because “other protophones” were not included in the prior study.

**Table 3:** **Summary table for odds ratio results**.

	**Squeal**		**Vocant**		**Growl**		**Other**	
**Predictions**	**ORs**	**95%CI**	**ORs**	**95%CI**	**ORs**	**95%CI**	**ORs**	**95%CI**
**0 mo**								
(A) Prot>cry in neutrality	24.6^***^	14.8	23.4^***^	9.4	22.6^***^	8.8	79.6^***^	21.5
		276.6		71.3		71.3		372.9
(B) Prot < cry in negativity	27.5^***^	7.8	25.5^***^	10.2	24.7^***^	9.5	127.9^***^	29.1
		108.1		77.4		77		562.4
(C) Prot>cry in positivity	18.6	0.73	5.6	0.32	6	0.31	36.1^*^	1.7
		472.2		96.9		118.2		781.2
**1 mo**								
(A) Prot>cry in neutrality	15.5^***^	4.6	25.7^***^	9.7	28.9^***^	10.1	25.4^***^	6.8
		54.2		75.3		91.5		102.8
(B) Prot < cry in negativity	21.6^***^	7.6	29^***^	12.5	35^***^	13.5	26.4^***^	7.8
		76.6		79.8		105.1		102.5
(C) Prot>cry in positivity	15.4^*^	1.7	7.8^*^	1.1	8.9^*^	1.1	4.4	0.27
		137.8		57.9		72.8		74.5
**2 mo**								
(A) Proto>cry in neutrality	25^***^	6.3	23.5^***^	8.7	40^***^	13.8	40.8^***^	11.0
		80.6		53.4		96.8		121.5
(B) Prot < cry in negativity	38.8^***^	13.9	38^***^	20.9	61.2^***^	20.9	60.2^***^	16.5
		104.1		178.8		178.8		220.9
(C) Prot>cry in positivity	45.9^*^	2.12	30.1^*^	1.8	26.0^*^	1.5	21.4	0.85
		994		491.7		461.0		541.5

* *p < 0.05*.

*** *p < 0.001*.

The data in Table 3 are based on means computed across the 5 coders for both vocal type and affect judgments. To interpret Table 3 consider an example: the odds ratio of 24.6 in the upper left cell (squeals > cry in neutrality at 0 months) means that squeals were 24.6 times more likely than cries to be associated with neutral facial affect at 0 months. The 95% confidence interval (95% CI) provides a measure of how reliable the obtained ORs were. For the prediction of squeals > cry in neutrality at 0 months, the CI includes the odds ratio of 24.6 but does not include 1.0 (which would represent equal odds of squeals and cry being neutral), and thus it can be concluded that the OR is significantly different from chance. The more distant 1 is from the CI presented in the table for any of the predictions, the more reliable the OR is in supporting the corresponding prediction.

In the left hand columns of Table 3 representing the results for squeals, vocants, and growls the odds ratios show that the three protophones conformed to the first two predictions (protophones > cry in neutrality, protophones < cry in negativity) with highly significant odds ratios (ORs > 1 and *p* < 0.001). Thus, starting from the first month of life, these three protophones were already differentiated from cries in two ways: they were significantly less negative than cries and significantly more neutral than cries [see ORs for predictions (A) and (B) across age and across the three protophone types], supporting hypothesis one.

For data on the third prediction for hypothesis one (protophones > cry in positivity), the results were less consistent, showing statistical support for the prediction at 1 and 2 months, but not at the youngest age, 0 months. However, it should be noted that assessment of the positivity prediction was hampered by very small sample size, owing to the fact that infants as young as these scarcely ever smile. In the data at 0 months, there were only eight protophones altogether coded as having positive facial affect, and consequently it may not be sensible to evaluate the positivity prediction at this age.

To assess the second hypothesis, we used the same odds ratio approach with reference to the right hand column of Table 3. The data show that “other protophones,” like the squeals, vocants, and growls, also conformed to the predictions of neutrality (A) and negativity (B) with ORs much higher than 1 and *p* < 0.001. Thus, the second hypothesis was confirmed for predictions A and B. For the positivity prediction (C) however, the results were mixed, and unexpected. At 0 months, “other protophones” conformed to the prediction significantly, but at 1 and 2 months, the results were not significant. Unlike the squeals, vocants, and growls, the “other protophones” did not significantly conform to the positivity prediction. Given the low number of positive expressions of affect in the data, we are inclined to be skeptical of all the outcomes on positivity.

The patterns of ORs in Table 3 represent the average across the five coders for vocal type and affect judgments. However, we also analyzed the data in terms of OR for each of the coders individually, and all of them showed statistically significant ORs consistent with predictions A and B for all protophones. Thus the pattern of greater the pattern of greater functional flexibility of protophones as opposed to cries applied to all 6 infants.

## Discussion

The purpose of the present study was to examine the emergence of functional flexibility in infant protophones across the first 3 months. We found that starting in the first month, all the protophone types demonstrated strong functional flexibility by showing significantly more neutral facial affect than cry and significantly less negative facial affect. The odds ratios used to illustrate the points showed highly significant conformity to predictions A (protophones > neutral in facial affect than cries) and B (protophones < negative in facial affect than cries). We anticipated functional flexibility to be discernible in the first 3 months in squeals, growls, and vocants as well as in the “other protophones,” and this finding supports both hypotheses that we evaluated. We also found that infant protophones were functionally flexible across all 3 months, being differentiated from cry at all the ages.

To interpret the results, we might consider the idea of protophones as a platform for development of speech, also serving to help indicate infants' affective/emotional state and condition of well-being (Oller and Griebel, 2006). Facial affect is coordinated in time with vocalization in infancy (Yale et al., 1999), so vocalization can also serve to draw attention to the expression of affect through the visual modality. It is important for infants to express negativity when they are in need of help and care through vocalizations (both cry and protophones can serve this function), and it is also important for them to express neutrality vocally when they are not in distress (cry cannot do that).

Although the 0–2 month old infants of the present study showed higher rates of negativity and lower rates of neutrality than the 3–11 month old infants of Oller et al. (2013), their protophones were associated with neutral facial affect very often, and significantly more often than cry was. Further the protophones of these 0–2 month-olds were associated with negative facial affect significantly less than cry. We reason that early protophones, though not as neutral as those produced by older infants, are useful in two ways: first to form a platform for speech development and second to display well-being and fitness to their caregivers. The high frequency of occurrence of facial neutrality in the protophones (60% of all the protophones were facially neutral in these 0–2 month olds) suggests a foundation for speech since it must be possible to produce all words and sentences in language with neutral affect. The finding of high frequency of neutrality in vocalization of infants in the first month contradicts a long tradition of belief that infants begin life able only to express negativity with the voice, with a distinct emphasis on crying is the vocal expression of the newborn (Wasz-Hockert et al., 1964; Truby and Lind, 1965; Wolff, 1969; Stark et al., 1975; Prechtl, 1984; Lester and Boukydis, 1992; Michelsson and Michelsson, 1999). Clearly, protophones are fully active in the first month of life and fully able to participate in expression of neutrality and presumably of a state of comfort and well-being.

The results on positive facial affect were mixed and can be viewed as somewhat predictable based on facts about infant development. We anticipated finding protophones to be more affectively positive than cries across age, but the odds ratio analyses showed that only the “other protophones” conformed to the prediction of positivity significantly at 0 months, while the three phonatory protophones conformed only at 1 and 2 months (Table 3). Since very young infants smile and laugh rarely until 3 or 4 months, the frequency of occurrence of positive facial expression was very low, and even so, some of the protophones conformed to prediction C with significant ORs.

The present study documents the emergence of functional flexibility in the first year and quantifies it in such a way that is comparable with results on older infants in Oller et al. (2013). For both studies, neutrality was dominant in the production of protophones and vocant was the most frequently occurring protophone type among all protophone types studied. In adult speech, neutral facial affect and normal phonation as it occurs in the production of vocants are also dominant. The importance of functional flexibility in vocalization has also recently been recognized in cross-species research, where the possibility of some degree of functional flexibility in vocalizations of our closest primate relatives is being pursued (Clay et al., 2015).

The results do not necessarily suggest that infants *intend* to associate vocal types flexibly with affective states. They may simply produce the protophones in whatever affective state they happen to be in. This possibility does not undercut the importance of functional flexibility because even non-volitional communication may form a foundation for later, more volitional communication.

We reason that the similarities found in protophones and adult speech are not a coincidence. It is important to recognize that infant protophones reveal many properties of speech in a simpler form. We used affect to determine communicative functions expressed by infants. Adults can also express communicative functions primarily through affect although there are many more options available to adults. For example, adults can simply state their communicative intentions directly: “Here is my prediction about publishability of your article.” This is as a statement that marks a possible communicative intent (i.e., illocutionary force) of prediction. Similarly, “I hereby criticize your choice of wording.” This is a sentence that could be used to express a criticism of our paper. Neither of these sentences could be produced by an infant, and neither of the communicative intents (prediction or criticism) could be specified by a young infant's actions. There are many illocutionary forces available to any mature speaker of a language that cannot be produced by an infant: stipulation, denial, explanation, reiteration, and so on. Any adult can use any word or sentence to express different illocutionary forces on different occasions, and this represents the pinnacle of functional flexibility. Language makes such complexities possible. The finding that even in the first month of life, human infants showed functional flexibility in the production of protophones suggests that this foundation for speech runs very deep in human nature. The fact that we observed protophones accompanying different facial affect types appears thus to indicate that functional flexibility of protophones is an important milestone on the path to the speech capacity.

## Author contributions

YJ coordinated the coders, designed the study, analyzed the data, and wrote the paper. DKO designed the study, analyzed the data, and wrote the paper.

## Funding

This work was primarily supported by two grants from the National Institutes of Health R01 DC011027 and R01 DC006099.

### Conflict of interest statement

The authors declare that the research was conducted in the absence of any commercial or financial relationships that could be construed as a potential conflict of interest.

## Footnotes

^1^“Other protophones” are distinguished from the three phonatory protophone types (i.e. squeals, growls, and vocants) because their sources of acoustic energy (Fant, 1960) are not at the glottis as in the three phonatory protophones, but in the supraglottal vocal tract: the lips, palate, tongue, and pharynx.
