# Supplementary material for: Emergence of Functional Flexibility in Infant Vocalizations of the First 3 Months
Source: Front Psychol. 2017 Mar 24;8:300. doi: 10.3389/fpsyg.2017.00300 (PMC5364184; doi:10.3389/fpsyg.2017.00300)
Supplement: Supplementary file 1 [file Table1.docx]

Supplementary Material

Emergence of Functional Flexibility

in Infant Vocalizations of the First Three Months

Yuna Jhang^1,3*^, D. Kimbrough Oller^1,2,3^

*** Correspondence:** Corresponding Author: [highyuna@gmail.com](mailto:highyuna@gmail.com)

**Supplementary Table.** Each cell represents the average number of utterances categorized by facial affect and vocal type across five coders.

| 0 mo | Facial Affect  Vocal Type | Can’t see | | | Negative | | | Neutral | | | Positive | |  |
| --- | --- | --- | --- | --- | --- | --- | --- | --- | --- | --- | --- | --- | --- |
|  | Cry | 4 | | | 128.2 | | | 4.4 | | | 0 | |  |
|  | Vocant | 12.8 | | | 221.8 | | | 186 | | | 8.4 | |  |
|  | Growl | 5.2 | | | 85 | | | 68.6 | | | 3.4 | |  |
|  | Squeal | 0.6 | | | 11 | | | 9.8 | | | 0.6 | |  |
|  | Other | 0.4 | | | 3.6 | | | 14.2 | | | 1.6 | |  |
| 1 mo | Facial Affect  Vocal Type | | Can’t see | | | Negative | | | Neutral | | | Positive | |
|  | Cry | | 0.8 | | | 75.6 | | | 4.2 | | | 1.2 | |
|  | Vocant | | 8.4 | | | 142.4 | | | 256 | | | 39.4 | |
|  | Growl | | 0.4 | | | 22.8 | | | 49 | | | 8.2 | |
|  | Squeal | | 0.6 | | | 12.2 | | | 14.2 | | | 4.6 | |
|  | Other | | 0.4 | | | 6.8 | | | 11.4 | | | 1.4 | |
| 2 mo | Facial Affect  Vocal Type | | | Can’t see | | | Negative | | | Neutral | | Positive | |
|  | Cry | | | 1.4 | | | 155.4 | | | 4.6 | | 0 | |
|  | Vocant | | | 1.4 | | | 261.4 | | | 212.2 | | 43.6 | |
|  | Growl | | | 1.2 | | | 38.2 | | | 53.4 | | 7 | |
|  | Squeal | | | 0 | | | 9.2 | | | 8 | | 1.6 | |
|  | Other | | | 0 | | | 9.2 | | | 12.8 | | 1.4 | |
