# Supplementary material for: Emergence of Functional Flexibility in Infant Vocalizations of the First 3 Months
Source: Front Psychol. 2017 Mar 24;8:300. doi: 10.3389/fpsyg.2017.00300 (PMC5364184; doi:10.3389/fpsyg.2017.00300)
Supplement: Supplementary file 2 [file Presentation1.pdf]

Supplementary Material

Emergence of Functional Flexibility

in Infant Vocalizations of the First Three Months

Yuna Jhang[1,3*], D. Kimbrough Oller[1,2,3]

*** Correspondence:** Corresponding Author: yjhang@memphis.edu

Supplementary Figures. The following example utterances are from one of the infants in the present study at 0 months of age, all the examples occurring within a 10 minute period. They illustrate that the three primary protophone types (Squeal, Vocant, Growl) are clearly present at 0 months, much as they are at older ages as illustrated in our prior work (see Oller et al. 2013, PNAS, Supporting Information Appendix)

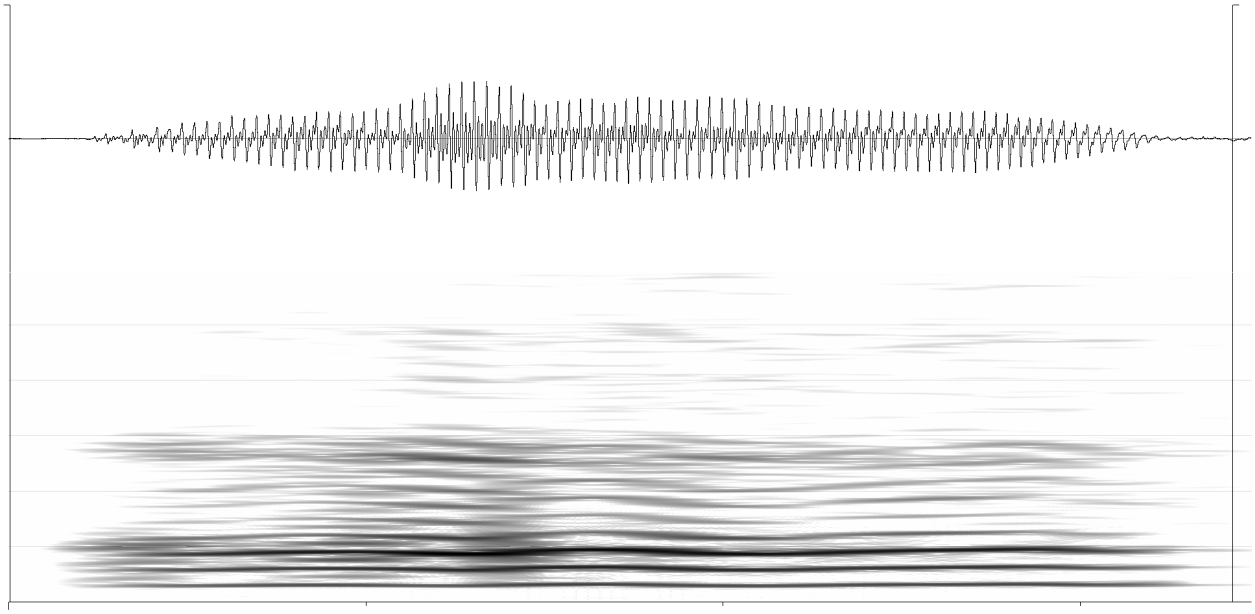

Figure SM1. A vocant at 0 months (display range=6 kHz, utterance duration=320 ms). The horizontal lines at equal intervals represent (0-6 kHz), and the first harmonic of the vocant along with pitch period analysis based on the waveform suggest f0 at 260-270 Hz. The auditory impression is of a sound produced in normal phonation, the same pattern of phonation found in most vocants produced by older infants. The audio example is Audio1: vocant.wav.

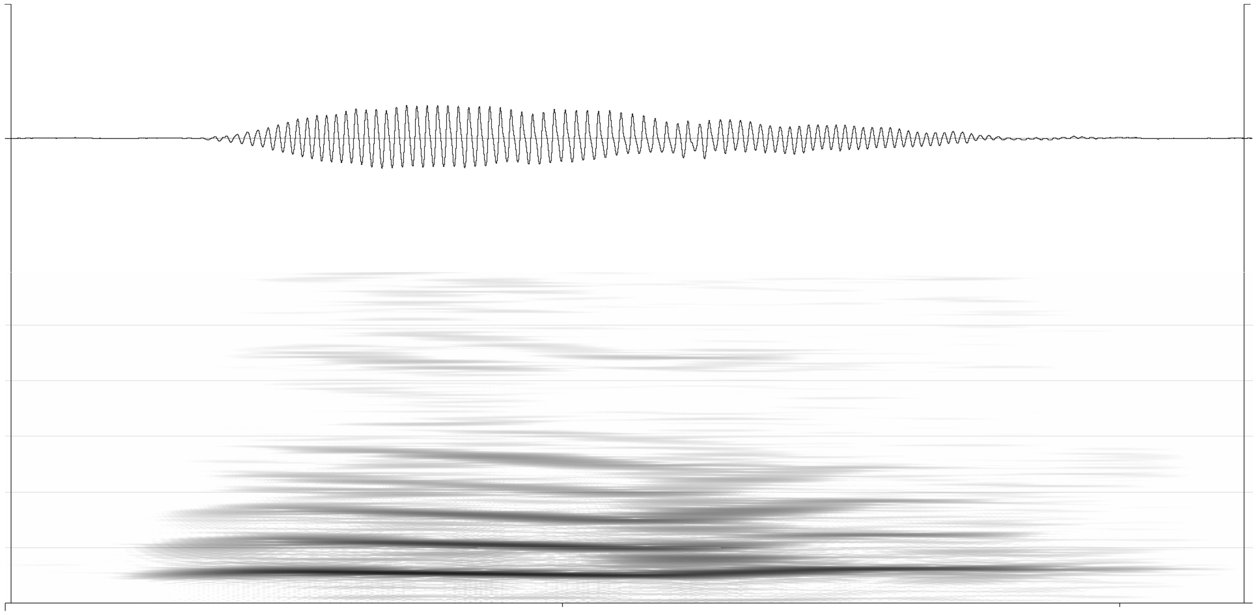

Figure SM2. A squeal at 0 months (display range=6 kHz, utterance duration=200 ms). The first harmonic of the squeal along with pitch period analysis of the waveform suggest f0 at 500-560 Hz, about twice as high as the f0 in the vocant, Figure SM1. The auditory impression is of a sound produced in loft (falsetto) register, the same way most squeals are produced in older infants. The audio example is Audio 2: squeal.wav.

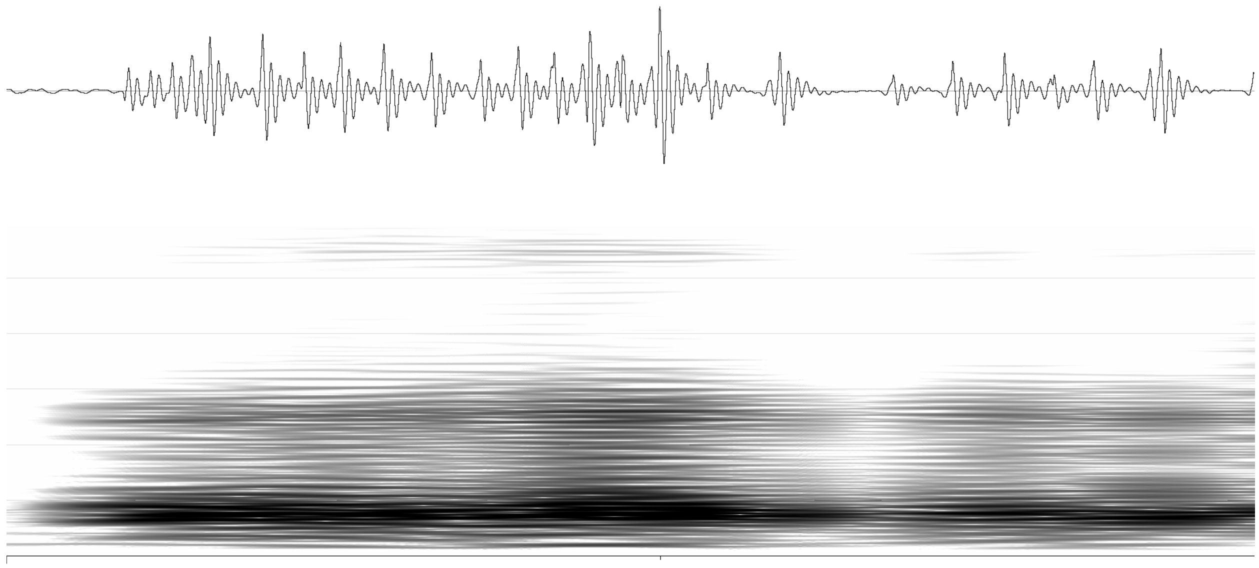

Figure SM3. A growl at 0 months (display range=6 kHz, utterance duration=200 ms). The first harmonic of the growl along with pitch period analysis of the waveform suggest f0 at 90-150 Hz, less than half as high as the f0 in the vocant, Figure SM1. The auditory impression is of a sound produced in pulse (fry) register with notable dysphonation, the same way most growls are produced by older infants. The audio example is Audio 3: growl.wav.
